# Deliberate paradigm shift in research in rare neurodevelopmental disorders

**DOI:** 10.1186/s13023-021-01885-3

**Published:** 2021-06-09

**Authors:** Jennifer M. Bain, Adel Ardalan, Sylvie Goldman

**Affiliations:** 1 Department of Neurology, Division of Child Neurology, Columbia University Irving Medical Center, 180 Fort Washington Avenue, 5th Floor, New York, NY 10032 USA; 2 Zuckerman Mind Brain Behavior Institute, Columbia University, 3227 Broadway, New York, NY 10027 USA; 3 Department of Neurology, Division of Cognitive Neuroscience, Columbia University Irving Medical Center, 622 West 168th Street, PH18-331, New York, NY 10032 USA

## Abstract

Diagnosis and management of children with rare neurodevelopmental disorders (RNDDs) are complex. The COVID-19 pandemic has forced us to rethink the research activities critical to improve our understanding and treatment of RNDDs, such as creating large international registries and developing natural history studies. In this communication, we reflect on our latest effort in conducting research remotely while providing support, education and feedback to families affected by a specific RNDD. Specifically, we advocate for a deliberate paradigm shift towards virtual family meetings as ecological platforms to enroll and assess individuals with rare disorders. Herein, we demonstrate that such a shift is crucial to substantially increasing geographical and age range coverage, which are essential for capturing the phenotypic variations in RNDDs. Finally, we call on the community to invest in building integrated technological platforms necessary for effective remote research activities, through standardization, collaboration and training.

## Introduction

A rare neurodevelopmental disorder (RNDD) is defined as a neurodevelopmental condition that affects fewer than 200,000 people. The diagnosis and management of children with RNDDs are complex for a variety of reasons, including limited information about these ultra-rare conditions and the prevalence of challenging behaviors. In addition, individuals with RNDDs and their families are often more susceptible to elevated anxiety and social seclusion. These challenges, in turn, make enrollment and research on RNDDs difficult to conduct and sustain.

As a result, comprehensive research programs are essential to refine methods of screening and diagnosis, and to develop targeted treatments for clinical trials. The natural history of many RNDDs needs to be described in detail before an intervention can be proposed that will impact the natural trajectory of such disorders. This calls for large international registries and uninterrupted natural history studies (NHS). Developing an encompassing natural history requires identification and enrollment of many individuals and their families, often necessitating an international quorum to increase the cohort size. Such enrollment is often challenging due to high cost for participation, language barriers, and time and traveling constraints. These logistical barriers can be further exacerbated by the behavioral challenges of patients with RNDDs.

The multitude of restrictions imposed by COVID-19 pandemic have significantly hindered the NHS of RNDDs and as a result, many research teams (as well as providers and educators) have rushed in to temporarily remedy the situation by moving their activities online, with many of them planning to go back to pre-pandemic practices as soon as the pandemic would be under control. Here, we argue that the RNDD research community should embrace a deliberate and concerted paradigm shift toward sustained online research activities for the following reasons: (1) Virtual family meetings could provide a more accessible, convenient and intimate way for individuals with rare disorders and their families, to participate in research studies; (2) These virtual meetings use more ecological platforms for assessment and treatment of individuals with RNDDs; and (3) Remote enrollment can substantially increase the age range and geographical coverage of the studies, which are vital for capturing the phenotypic spectrum of RNDDs.

Here, we report on our most recent effort in conducting research remotely to provide support, education and feedback to families affected by a specific RNDD and share the lessons we learned from our experience. Due to pandemic-related restrictions our annual in-person family meeting could not be conducted as usually planned; no in-person data could be collected, and no social gathering could be organized around lectures and lunches. However, as the continuity of care and research was paramount, we decided to shift our family meeting to a virtual format to avoid a disruption in our natural history study and other research efforts. Our study was unique in its depth (detailed behavioral and computational studies), breadth (natural history, physical and occupational therapy, gait analysis) and scale (geographical coverage and age range).

## Study outline

Our team has focused on understanding the developmental trajectory of individuals with *HNRNPH2* gene variants associated with a neurodevelopmental disorder [1, 2]. *HNRNPH2*-related disorder (OMIM 300,986 Mental retardation, X-linked, syndromic, Bain type; MRXSB) is a newly described neurodevelopmental disorder caused by pathogenic variants in the *HNRNPH2* gene [1, 2]. *HNRNPH2* is located on the X chromosome and encodes Heterogeneous Nuclear Ribonucleoprotein H2 (HNRNPH2), a ubiquitously expressed RNA-binding protein that primarily functions to regulate the alternative splicing of pre-mRNA [3, 4]. *HNRNPH2* is one of several HNRNPs that are associated with a neurodevelopmental disorder [5, 6]. Bain et al. initially described *HNRNPH2*-related disorder in 6 females with a similar neurodevelopmental phenotype including global developmental delay/ intellectual disability, severe motor impairment, musculoskeletal problems and neuropsychiatric diagnoses [5]. We recently expanded on the phenotype including 33 individuals and a more detailed clinical description of the disorder [6].

**Fig. 1:**
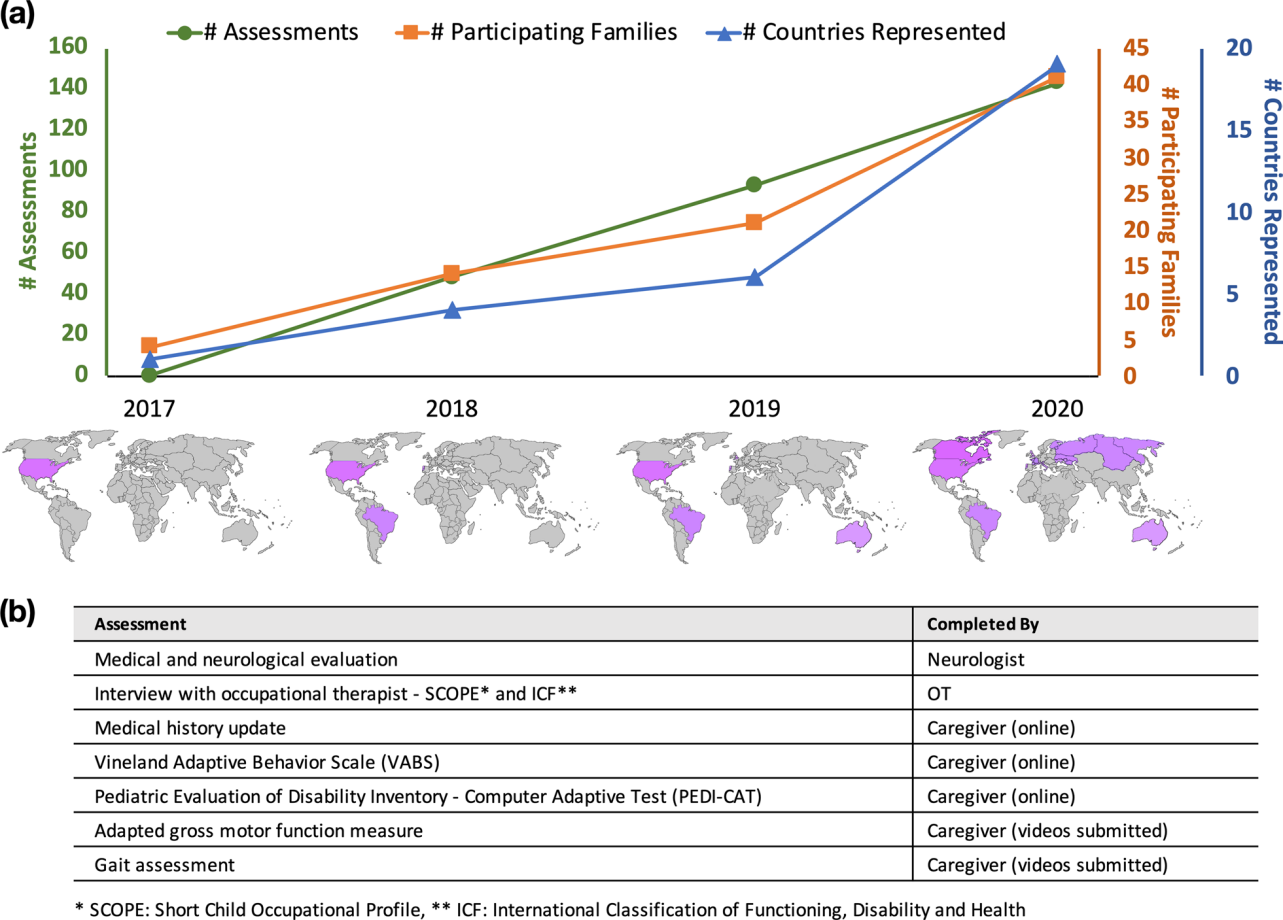
**a** Increasing number of assessments (green circles), number of participating families (orange square) and number of countries represented (blue triangle). **b** List of completed assessments administered during the virtual family meeting and responders

Since the initial description in 2016, we have aligned with the parent-run non-profit organization Yellow Brick Road Project (YBRP) to increase awareness about the importance of participating in a natural history study (Fig. 1a). Within a year of initial publication, we started a natural history study, and we continue to enroll new subjects monthly (www.hnrnph2.com). Participants were referred to this study (ClinicalTrials.gov NCT03492060) if they were diagnosed with pathogenic or likely pathogenic variants in Heterogeneous Nuclear Ribonucleoprotein H2 (*HNRNPH2)* using ACMG/AMP criteria, largely identified via clinical exome sequencing. The study was approved by the ethic committee of the Columbia University Institutional Review Board and informed consent was obtained from all caregivers or legal guardians. Our research protocol and data results is available upon request to the authors for our Natural History Study and Virtual Shift Protocol.

In close collaboration with YBRP, we held annual family meetings in 2018 and 2019, during which time our enrollment increased (Fig. 1a). In-person assessments included neurological, medical and orthopedic examinations as well as physical and occupational therapy evaluations. At the last in-person meeting, in New York City which was at a local conference center with guest rooms and leisure spaces like a gym and pool, families were able to schedule their research evaluations as well as plan for rest time and breaks when needed. There was also a dedicated space for care and play time provided for affected individuals to be cared for while parents and families participated in lectures and meetings with other research activities.

**Fig. 2:**
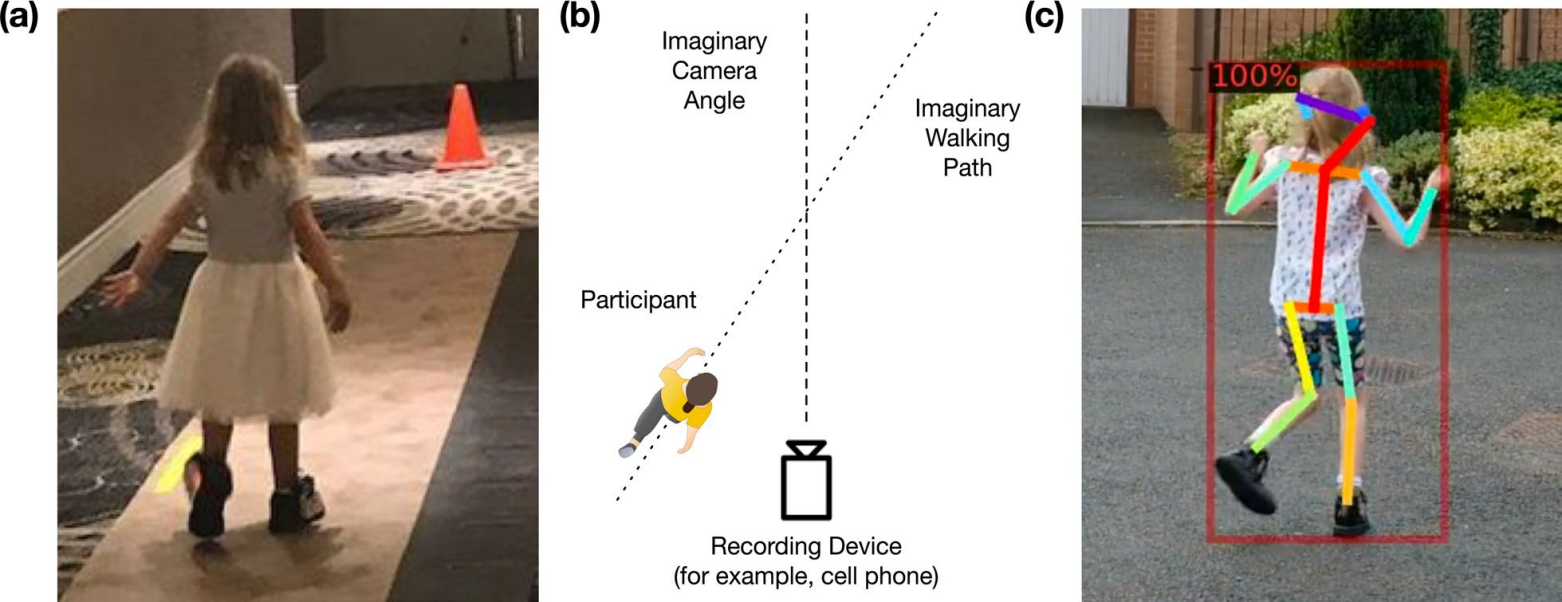
**a** Toddler girl walking on a sensor-walkway mat wearing instrumented sneakers. **b** Instructions for gait videos recording. **c** AI based pose estimation extracted from home videos

Given the significant motor impairments in this group, characterizing the motor delays and dysfunction has been a major focus of our research team and we sought to capture data from various sources. At the start of each annual meeting, each family completed the Pediatric Evaluation of Disability Inventory Computer Adaptive Test (PEDI-CAT), which is a caregiver‐reported outcome measure that evaluates functional domains including daily activities, mobility, social/cognitive and responsibility [7–9]. The instrument was designed to evaluate individuals ages birth to 21 years old and has been validated for children with disabilities over a variety of diagnoses including autism spectrum disorder, Rett Syndrome and spinal muscular atrophy [10, 11]. After completion of the PEDI-CAT, each participant was evaluated by a trained physical therapist to measure motor skills using the gross motor function measure (GMFM-88). The GMFM-88 is an in person 88-item evaluative tool that was initially designed and validated to measure gross motor function in children with cerebral palsy [12]. The GMFM‐88 assesses a wide spectrum of gross motor activities including the following five dimensions: (A) lying and rolling, (B) sitting, (C) crawling and kneeling, (D) standing, and (E) walking, running, and jumping. Each item is scored on a 4‐point scale with a maximum total raw score of 256 points and can be used into adulthood. Items from the GMFM-88 have been used to develop scales to measure motor function in Down Syndrome [13], spinal muscular atrophy [14] and even neurodevelopmental disorders such as Rett Syndrome [15]. We have previously shown strong correlation with the parent-report PEDI-CAT and clinician-administered GMFM (publication under review). Lastly, given that gait difficulties were identified as an intrusive symptom in this disorder by families, gait analysis was studied using insole technology and gait mat (Fig. 2a) [16]. The protocol of our natural history focused on standardized neurological examination and physical and occupational therapists’ evaluation. Further, cognitive level was assessed using the Vineland Adaptive Behaviors Scale rather than formal neurocognitive testing which may represent a limitation.

During this global pandemic, we sought to carry on with our NHS, with concern that the loss of a single year worth of data and interactions with the families would be detrimental to the progress of the study. In lieu of our regular in-person meeting, we worked closely with YBRP to shift to a virtual meeting, including one-on-one interviews, virtual video and questionnaire data collection and online family gatherings (Fig. 1b). In addition to data collection, the family meeting itself included 3 days of virtual conferences, roundtable discussions and social gatherings. Prior to the meeting, we provided a detailed illustrated instruction manual for scheduling video visits with the child neurologist and occupational therapy team, as well as how to collect and send behavioral data and fill out questionnaires. For the gross motor physical therapy assessment, we chose 23 items from the GMFM-88 instrument for individuals to perform at home which covered the main gross motor domains and captured both floor and ceiling effects of the participants’ motor profile. Families were instructed to videotape the motor tasks and upload them via online databanks. We offered individual physical therapy sessions for those families who needed further instructions or support to complete the assessment. In lieu of using in-sole shoe sensors with gait mat (Fig. 2a), we asked families to videotape specific gait tasks using our illustrated instruction checklist for later artificial intelligence (AI)—based pose estimation [17]. Figure 2b shows an example from the gait data collection instructions and Fig. 2c shows a sample result of extracting gait information using AI.

In addition to the gross motor and gait evaluations, we sought to further investigate fine motor deficits and sensory issues. This year’s assessment included a qualitative videotaped interview to complete the Short Childhood Occupational Profile (SCOPE) [18]. The OT team then combined the data from this interview with their completion of other standardized measures such as the Vineland Adaptive Behavior Scale 3rd Edition and Behavior Assessment System for Children 3^rd^ Edition to assign each subject an ICF score [19, 20]. The International Classification of Functioning, Disability and Health, known more commonly as ICF, is a classification of health and health-related domains [21]. This evaluation will be utilized for a deeper understanding of the occupational therapist needs by the group, as well as providing feedback to the family for further clinical recommendations.

**Fig. 3:**
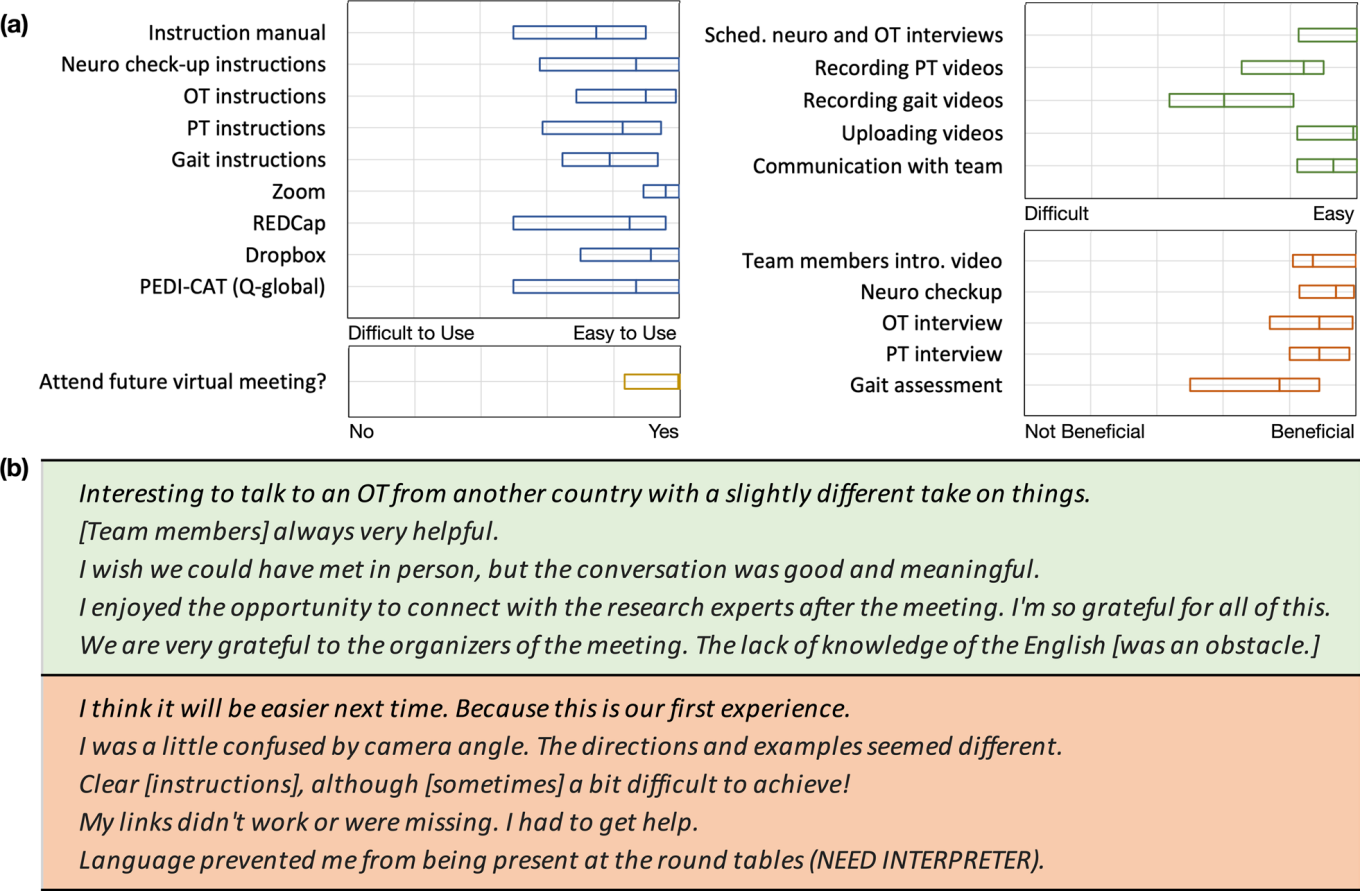
**a** Summary of the survey results by question type. **b** Examples of positive feedback (green) and issues (orange) raised by participating families

At the conclusion of the family meeting, we held a virtual focus group to gather families’ feedback on the process, as well as to reinforce the sense of community and continuity of care between the research team and family organization. We then distributed a survey to evaluate families’ satisfaction, identify specific issues and to inform future meetings (Fig. 3). Most of the feedback was positive regarding the experience of participants in a virtual meeting and many endorsed a positive response about future engagement with a virtual setting.

## Obstacles and disadvantages

One limitation of performing the natural history study in the virtual paradigm was the limited validity in telehealth cognitive testing. Since the start of the pandemic the lack of validity of remote cognitive assessment in children is a central debate in the field of neuropsychology. In order to mitigate this issue, we have followed the recommendations of the profession and administered the Vineland Adaptive Behavior Scale-Parents Interview to document cognitive changes for this year [22, 23].

Another limitation of this virtual platform was the loss of intimate connection in meeting families, clinicians and researchers during an in-person event. As noted in some of the qualitative feedback from families, these annual meetings often brought a deep feeling of belonging and an opportunity to learn from experts and other families. In the end, however, most families did report an overall positive experience and a strong desire to move forward and participate in future meetings as their way to contribute to scientific progress for their own child and the community of RNDD.

In regard to the technical setup, one obstacle in data collection was the challenge to obtain videos suitable for offline gait analysis. The current state of the art in pose estimation from video recordings remains sensitive to various parameters such as angle, lighting, occlusions, etc. Although we collected high-quality videos and were able to generate pose estimation data by providing detailed instructions and feedback to the caregivers on their initial recordings, they often had to repeat the recording to meet our criteria. With the advances in pose estimation technology, we expect that future data collection will be easier and more efficient. We also plan on using parents’ feedback to revise our instructions to make them clearer for higher quality standard.

Lastly, while most families reported small technical difficulties using the virtual platform, we are aware that several families were unable to participate due to limited access to video collection methods, computer systems or high-speed internet connections. Functional level of families to be able to read and follow the instructions provided to families in the instruction manual and use the technology needed for virtual meetings. There were also other families who are unable to provide care to their family member with special needs while also participating in the meeting itself. We also recognize the literacy and language barriers, as well as difficulties for a subset of families to be available for full days of meeting while caring for their child or young adults with special needs.

## Lessons learned

Based on the results from the focus group and the survey, we are highlighting the following points which we think will be useful for the RNDD research community to continue to provide high-quality care and study of individuals and families with RNDD while adapting to the new conditions.

- Virtual family meetings provide an ecological and intimate platform for valid assessment and treatment of individuals with rare disorders.
- Patients and their caregivers appeared comfortable and not pressured while performing the tasks in their homes.
- Although the families expressed grief for the lost sense of community and care they felt during in-person family meetings, they were happy to get a chance to contribute to the study, check in with researchers, practitioners and other families during these hard times, as well as receive information and feedback about their children’s progress.

Thanks to this paradigm shift, we were able to substantially increase the geographical coverage of our meeting. As shown in Fig. 1a, the number of international families who participated in the meeting tripled compared to the previous year. This was mostly due to reduced cost of participation, easier access, and wider reach of the recruitment effort. Moreover, we were able to extend the overall data collection time and thus avoid schedule conflict with other conferences or responsibilities within this short time period.

A wider geographical coverage is vital within RNDDs in order to include wider phenotypic variations, extend enrollment and care to under-privileged families as well as those reluctant to travel with their family member. While we were able to include a wider geographic area and collect more phenotypic variability, we were unable to demonstrate engagement with more under-privileged families. Future endeavors should seek to demonstrate the effective reach of such studies to individuals and families with currently limited access to high-speed internet and video capability. We are hopeful that the emergence of new internet technologies (e.g. recent World Mobile Group projects in Tanzania) and low-cost cameras (e.g. on affordable cell phones) would enable more under-privileged families to join future studies. Altogether, this experience helped us and the scientific community to practice how to build stronger and more global links with families.

We recognize that the integration of new technological tools requires effort to increase standardization, unification and training. Indeed, the research team and the participating families mentioned a lack of standardized streamlined technological platforms to encompass all planning, training, data collection and analysis that would facilitate a unified framework. The use of a series of tools for each assessment made participation more difficult and time-consuming for families.

In conclusion, in the era of precision medicine for the treatment of rare disorders, it is imperative and feasible to shift the research paradigm and continue gathering critical data. While a complete shift to virtual platform may still be complex, we suggest there is a need to incorporate data collected from this medium to complement in-person evaluations. We argue that this approach is essential to thrust robust and rigorous clinical trials and support families in dire need during these stressful times.

Resources available for families affected by rare diseases in the setting of the COVID19 pandemic and for researchers include:

- https://autismsciencefoundation.org/covid-19-resources/
- https://rarediseases.org/covid-19/
- https://globalgenes.org/2020/03/20/supporting-the-rare-disease-communityduring-covid-19/
- https://www.rarechromo.org/covid19update/

## Data Availability

We will provide our protocol upon request to the authors.

## Abbreviations

NHS: Natural history study
GMFM: Gross motor function measure
HNRNPH2: Heterogeneous Nuclear Ribonucleoprotein H2
ICF: International Classification of Functioning, Disability and Health
PEDI-CAT: Pediatric Evaluation of Disability Inventory Computer Adaptive Test
RNDD: Rare neurodevelopmental disorders
YBRP: Yellow Brick Road Project
SCOPE: Short Childhood Occupational Profile
